# Characterization of the complete mitochondrial genome of the hybrid grouper *Epinephelus akaara* ♀ × *Epinephelus tukula* ♂, with its phylogenetic analysis

**DOI:** 10.1080/23802359.2019.1673678

**Published:** 2019-10-04

**Authors:** Zhentong Li, Yongsheng Tian, Linlin Li, Yuping Wu, Jingjing Zhang, Ziqi Li, Linna Wang, Meiling Cheng, Zunfang Pang, Wenhui Ma, Jieming Zhai

**Affiliations:** a Key Laboratory of Sustainable Development of Marine Fisheries, Ministry of Agriculture and Rural Affairs, Yellow Sea Fisheries Research Institute, Chinese Academy of Fishery Sciences, Qingdao, China;; b College of Fisheries and Life Science, Shanghai Ocean University, Shanghai, China;; c Laboratory for Marine Fisheries Science and Food Production Processes, Qingdao National Laboratory for Marine Science and Technology, Qingdao, China;; d Chinese Academy of Agricultural Sciences, Beijing, China;; e College of Fisheries and Life Science, Dalian Ocean University, Dalian, China;; f Laizhou Mingbo Aquatic Co., Ltd, Yantai, China

**Keywords:** *Epinephelus akaara* ♀ × *Epinephelus tukula* ♂, mitochondrial genome, phylogenetic analysis

## Abstract

*Epinephelus akaara* ♀ × *Epinephelus tukula* ♂ is an economically important fish. The mitochondrial genome of the hybrid grouper had a double-stranded DNA molecule with the length of 16,928 bp and consists of 13 protein-coding genes, 22 tRNA genes, 2 rRNA genes, and a control region. The gene composition of the hybrid grouper mitochondrial genome was similar to that of most other vertebrates. Furthermore, phylogenetic analysis by maximum-likelihood (ML) method, based on the nucleotide sequences of 13 protein-coding genes, showed that the hybrid grouper has the closer relationship to *Epinephelus akaara* and confirmed that the mitochondrial genome is maternally inherited.

*Epinephelus akaara* and *E. tukula*, both belonged to Perciformes, Serranidae, Epinephelinae. *Epinephelus akaara*, commonly named as Hong Kong grouper, with the merit of high-quality food and abundant nutrients (Xie et al. 2016). *Epinephelus tukula*, also named as potato grouper, with the merit of fast growth (Craig et al. 2011). Hybridization commonly used in fish breeding for it allows for a combination of advantageous traits from different species (Cheng et al. 2019). The hybrid grouper was obtained by artificial insemination, taking *E. akaara* as female parent and *E. tukula* as male parent. Considering the hybrid has a great potential economic value and its genetic characteristics remain poorly understood. Here, we published the complete mitochondrial genome of *E. akaara* ♀ × *E. tukula* ♂ by using the next-generation sequencing techniques strategy. The specimen was collected from Laizhou Mingbo Aquatic Co., Ltd., Shandong, China (37°25′28.26″N 120°0′44.32″E). Samples stored in a −80 °C refrigerator with accession number 20190627AT01. Extraction of total genomic DNA using phenol-chloroform method.

The complete mitochondrial genome of *E. akaara* ♀ × *E. tukula* ♂ is 16,928 bp in length (GeneBank accession no.MN337034). The gene composition of mitochondrial genome of the hybrid was the same as that of most vertebrates, including 13 protein-coding genes, 22 tRNA, 2 rRNA, and a control region. The overall base composition of the hybrid offspring was 28.7% A, 27.8% C, 16.1% G and 27.4% T, with a slight (A + T) bias of 56.1% and AT-skew (0.024), and GC-skew (−0.265). Most of the genes were encoded on the heavy strand but ND6 and eight tRNA genes [Gln, Ala, Asn, Cys, Tyr, Glu, Pro, Ser (UCN)]. There are three kinds of initiation codons in the protein-coding genes, COX1 start with GTG and ATP6 start with CTG and remains were ATG. Three types of stop codons were taken in the protein-coding genes, including TAA (ND1, COX1, ATP8, ATP6, ND4L, ND5, ND6), TA (ND2, COX3), T (COX2, ND3, ND4, CYTB). The 22 tRNA genes were interspersed among the mitochondrial genome, ranging from 67 to 76 bp in length. 12S rRNA and 126 rRNA were located between the tRNA^Phe^ and tRNA^Leu(UUR)^ and separated by the tRNA^Val^. The control region with a rich A + T content (70.88%) was 1226 bp in length, located between tRNA^Pro^ and tRNA^Phe^.

The phylogenetic position of the hybrid grouper *E. akaara* ♀ × *E. tukula* ♂ was confirmed using the ML method (Kumar et al. 2016) based on the concatenated nucleotide sequences of 13 PCGs with other 30 Epinephelinae species. The results showed that *E. akaara* ♀ × *E. tukula* ♂ forms a clade with *E. akaara* with strong bootstrap support (Figure 1), which demonstrates that the mitogenome is maternally inherited. In addition, it will further enrich the mitogenome database of Epinephelinae, and the phylogenetic relationships of species in Epinephelinae will become clearer.

**Figure 1.**
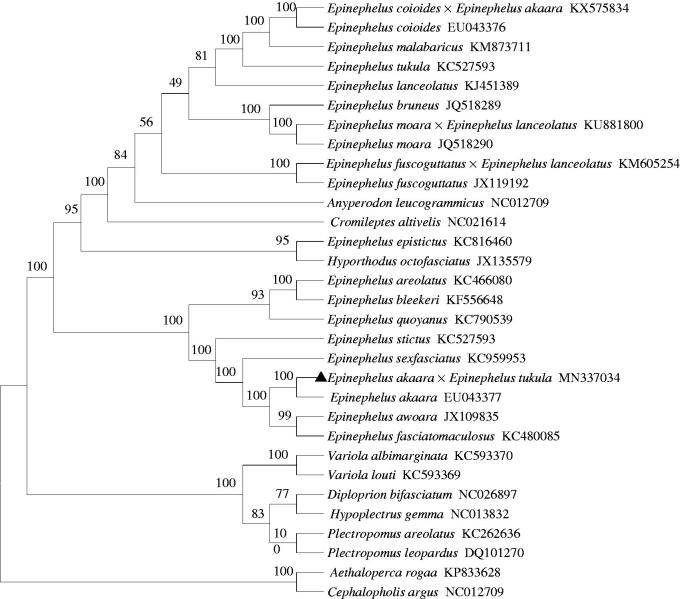
The ML phylogenetic tree of 31 Epinephelinae species. Numbers on each node are bootstrap values of 1000 replicates.
